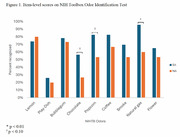# Are SuperAgers Super‐Sniffers? Performance on the NIH Toolbox Odor Identification Test in cognitive SuperAgers

**DOI:** 10.1002/alz70857_106245

**Published:** 2025-12-25

**Authors:** Carolina S Hernández, Kathryn A LaFroscia, Deborah Zemlock, Emily H Ho, Richard C. Gershon, Changiz Geula, Marsel Mesulam, Molly A Mather, Tamar Gefen, Sandra Weintraub

**Affiliations:** ^1^ Northwestern University Feinberg School of Medicine, Chicago, IL, USA; ^2^ Mesulam Center for Cognitive Neurology & Alzheimer's Disease, Chicago, IL, USA

## Abstract

**Background:**

Decline in the sense of smell has been associated with memory decline and is thus a potential early diagnostic marker for underlying Alzheimer's disease. However, it is unknown whether superior memory performance in aging is associated with advantage in olfaction. The Northwestern University SuperAging Program (NUSAP) studies a rare cognitive aging phenotype (i.e. “SuperAgers”) with exceptionally preserved episodic memory abilities above age 80. The aim of the current project is to determine whether SuperAgers perform better on a test of odor identification than same‐age controls with normal‐for‐age memory.

**Method:**

Sixty‐one participants enrolled in the Northwestern ADRC and NUSAP completed the NIH Toolbox Odor Identification Test (NIHTB‐OIT), either as part of their participation in another study, ARMADA (see Weintraub et al., 2022), or during an annual UDS study visit [(M_age_ = 87.20, SD = 0.49); 62% Female; 89% non‐Hispanic white; (M_education_ = 16.95, SD = 0.49)]. SuperAgers scored at least within the average range on the RAVLT Delayed Recall for individuals in their 50s‐60s at the time of NIHTB‐OIT administration; Normal‐Agers scored within the average range for their own age group. The NIHTB‐OIT asks participants to smell nine scratch‐and‐sniff cards and identify the smell from a visual array of four options. Independent samples t‐tests and chi‐square tests compared scores between groups for total and item‐level scores, respectively.

**Result:**

SAs and NAs did not differ in age, years of education, sex, or race/ethnicity. Total OIT score was higher in SAs compared to NAs and trended toward significance (*p* = 0.063). Item‐level scores were available for a subset of the total group. Across both groups, Play‐Doh was most unidentifiable. Interestingly, compared to NAs, SAs were significantly more likely to identify natural gas (*p* <0.01), and differences trended toward significance for chocolate (*p* = 0.070) and popcorn (*p* = 0.052; Figure 1).

**Conclusion:**

Preliminary findings based on NIHTB‐OIT performances suggest that SuperAgers demonstrate stronger odor identification abilities compared to their cognitively average peers, possibly due to the integrity of the memory‐related limbic system that includes the olfactory system. Further exploration of olfaction and other sensory functions in SuperAging may augment the biological signature of exceptional memory performance in old age.